# Anatomical distribution of CGRP-containing lumbosacral spinal afferent neurons in the mouse uterine horn

**DOI:** 10.3389/fnins.2022.1012329

**Published:** 2022-09-28

**Authors:** Kelsi N. Dodds, Melinda A. Kyloh, Lee Travis, Mack Cox, Tim J. Hibberd, Nick J. Spencer

**Affiliations:** College of Medicine and Public Health, Flinders Health and Medical Research Institute, Flinders University of South Australia, Bedford Park, SA, Australia

**Keywords:** uterus, pain, CGRP, sensory innervation, spinal ganglia

## Abstract

Sensory stimuli from the uterus are detected by spinal afferent neurons whose cell bodies arise from thoracolumbar and lumbosacral dorsal root ganglia (DRG). Using an *in vivo* survival surgical technique developed in our laboratory to remove select DRG from live mice, we recently quantified the topographical distribution of thoracolumbar spinal afferents innervating the mouse uterine horn, revealed by loss of immunoreactivity to calcitonin gene-related peptide (CGRP). Here, we used the same technique to investigate the distribution of lumbosacral uterine spinal afferents, in which L5-S1 DRG were unilaterally removed from adult female C57BL/6J mice (*N* = 6). Following 10–12 days recovery, CGRP immunoreactivity was quantified along the length of uterine horns using fluorescence immunohistochemistry. Relative to myometrial thickness, overall CGRP density in uterine tissues ipsilateral to L5-S1 DRG removal was reduced compared to the DRG-intact, contralateral side (*P* = 0.0265). Regionally, however, myometrial CGRP density was unchanged in the cranial, mid, and caudal portions. Similarly, CGRP-expressing nerve fiber counts, network lengths, junctions, and the proportion of area occupied by CGRP immunoreactivity were unaffected by DRG removal (*P* ≥ 0.2438). Retrograde neuronal tracing from the caudal uterine horn revealed fewer spinal afferents here arise from lumbosacral than thoracolumbar DRG (*P* = 0.0442) (*N* = 4). These data indicate that, unlike thoracolumbar DRG, lumbosacral spinal afferent nerves supply relatively modest sensory innervation across the mouse uterine horn, with no regional specificity. We conclude most sensory information between the mouse uterine horn and central nervous system is likely relayed *via* thoracolumbar spinal afferents.

## Introduction

The uterus is a major organ of the female reproductive tract that is richly innervated by sensory nerve fibers. In addition to detecting various innocuous and noxious stimuli, uterine sensory nerves can release neuropeptides in an efferent manner to modulate uterine contractions (Klukovits et al., 2004), blood flow (Gangula et al., 2003), and other regulatory processes critical for reproductive success (Collins et al., 2002). Earlier studies in rodents demonstrated that sensory innervation is primarily supplied to the uterus by spinal afferent neurons, whose cell bodies lie adjacent to the spinal cord in dorsal root ganglia (DRG) (Berkley et al., 1988; Herweijer et al., 2014). Spinal afferents convey sensory information between the uterus (and indeed other visceral organs) and the central nervous system through dual thoracolumbar and lumbosacral DRG pathways, *via* the hypogastric and pelvic nerves, respectively (Brierley et al., 2018). Few afferent fibers from nodose ganglia may also innervate the uterus, *via* the vagal nerves (Ortega-Villalobos et al., 1990; Collins et al., 1999; Dodds et al., 2021).

An enduring question in visceral sensory physiology is why vertebrates have evolved with two distinct populations of DRG innervating the same target organs (Christianson et al., 2006; Kyloh et al., 2011; Harrington et al., 2019). For the uterus, it is unclear whether the density and distribution of thoracolumbar and lumbosacral spinal afferents differ along its length, and what the relative contributions of these specific populations are to reproductive physiology and behavior. Our understanding thus far has been inferred from studies using hypogastric or pelvic nerve transection (Higuchi et al., 1987; Burden et al., 1990; Boyd et al., 2009; Mackay et al., 2009). However, the precise role of spinal afferents may be confounded by the presence of both sensory and motor pathways within these major nerve trunks. This identifies need for sensory- and pathway-specific ablation approaches that can probe the anatomy and function of distinct DRG populations.

To address this, we recently developed a survival surgery in mice where subsets of DRG are removed *in vivo* (Kyloh et al., 2022). Given the largely ipsilateral innervation to each half of the female rodent reproductive tract (Klein and Burden, 1988; Nance et al., 1988), deafferentation in the uterine horn due to unilateral DRG removal could be compared against the contralateral side. In that study, thoracolumbar (T13-L2) DRG ablation revealed most thoracolumbar spinal afferents (∼60%) containing the sensory neuropeptide, calcitonin gene-related peptide (CGRP), innervate the cranial end of the mouse uterine horn, while a smaller proportion (∼45%) supply the caudal region (Kyloh et al., 2022). These data support the topographical arrangement suggested for substance P-containing thoracolumbar spinal afferents in the rat uterine horn (Nance et al., 1988), which was also deduced from functional studies on the rat female reproductive tract (Berkley et al., 1988, 1990, 1993).

Here, lumbosacral (L5-S1) DRG were unilaterally ablated using our DRG removal technique. The residual distribution of CGRP-expressing nerve fibers in the mouse uterine horn was assessed to infer the pattern of lumbosacral (L5-S1) spinal afferent innervation. We hypothesized major loss of CGRP immunoreactivity from the caudal end, as this is the suggested target of lumbosacral innervation in the rat uterine horn (Berkley et al., 1988, 1990, 1993; Nance et al., 1988). Unexpectedly, only ∼10% depletion of CGRP-expressing nerve fibers occurred across the mouse uterine horn—evenly distributed along the cranial-caudal axis. Retrograde neuronal tracing from the caudal uterine horn of mice supported these results, revealing that fewer spinal afferents arise from lumbosacral than thoracolumbar DRG in this uterine region.

## Methods

### Animals

Nulliparous female C57BL6/J mice (*N* = 10), 3–6 months-old weighing 20.3 ± 2.3 g, were obtained from the College of Medicine and Public Health Animal Facility of Flinders University of South Australia. All animal use in this study was approved by the Animal Welfare Committee of Flinders University of South Australia (approval #966/19), and all protocols carried out in strict accordance with the National Health and Medical Research Council (NHMRC) Australian code for the care and use of animals for scientific purposes (8th edition, 2013) and recommendations from the NHMRC Guidelines to promote the wellbeing of animals used for scientific purposes (2008).

### *In vivo* removal of lumbosacral dorsal root ganglia

#### Surgery

This procedure has recently been described in detail (Kyloh et al., 2022). Mice (*N* = 6) were anesthetized with inhaled isoflurane (induced at 4%; maintained at 1.5–2% in 1 L/min O_2_) and administered s.c. 0.05 mg/kg buprenorphine (Temvet; Troy Laboratories; Glendenning; NSW, Australia). A ∼20 mm vertical midline incision was made along the dorsal skin to reveal the lumbosacral vertebrae. A further incision was made unilaterally into the skeletal muscle overlaying the DRG, and the animal’s left-hand L5-S1 DRG exposed. Dorsal roots bridging these DRG and the spinal cord were then transected, followed by complete removal of the DRG. The incision sites were irrigated with 0.5% bupivacaine (Marcain; AstraZeneca; North Ryde, NSW, Australia) and sutured closed. Animals were then administered a second s.c. dose of 0.05 mg/kg buprenorphine as well as s.c. antibiotics 100 mg/kg ampicillin (Alphapharm; Sydney, NSW, Australia) and 10 mg/kg baytril (Bayer; Pymble, NSW, Australia). Following withdrawal of anesthesia, animals recovered on a heat mat with 1 L/min O_2_ until fully mobile, then returned to their home cage. Post-operatively, animals received 0.1 mg/kg oral buprenorphine (Schering Plough; Macquarie Park, NSW, Australia) in hazelnut paste (Nutella^®^; Ferrero; Lithgow, NSW, Australia) daily for 72 h.

#### Immunohistochemistry

Following 10–12 days recovery, animals were deeply anesthetized with isoflurane (5% in O_2_) and exsanguinated. Complete L5-S1 DRG removal was confirmed postmortem and estrous cycle stage noted *via* vaginal smear cytology, as described in Dodds et al. (2015). Uterine horns were removed (from between the uterine cervix and internal bifurcation) and placed into Sylgard™-lined (Dow-Corning #3097358-1004; Midland, MI, United States) glass Petri dishes containing phosphate-buffered saline (PBS; 0.1 M), then secured to the base with entomology pins. Excess tissue was discarded, and the uterine horns sliced longitudinally along the mesometrial border. Each uterine horn was then pinned as a flat sheet preparation and fixed overnight with 4% paraformaldehyde (in PBS; pH 7.2). The endometrium was dissected free, and the remaining myometrial preparations cleared with dimethyl sulfoxide and blocked for 1 h with 1% bovine serum albumin/5% normal donkey serum/1% triton X-100. Preparations were then incubated in rabbit anti-CGRP polyclonal antibody diluted in block solution (1:2,000 from neat antiserum; product #T-4032; Peninsula Laboratories International, Inc.; San Carlos, CA, United States; RRID:AB_518147) for 2 nights, followed by incubation in donkey anti-rabbit CY5 antibody (7.5 μg/mL; product #711-175-152; Jackson ImmunoResearch Laboratories, Inc.; West Grove, PA, United States; RRID:AB_2340607) for 4 h. Finally, preparations were equilibrated with 50%, 70%, and 100% carbonate-buffered glycerol, then mounted, circular myometrium facing uppermost, on glass slides with 100% carbonate-buffered glycerol (pH 8.6). PBS washes were performed between all antibody incubation steps, as appropriate. All solutions were diluted in PBS/0.1% sodium azide unless otherwise specified and all incubation steps carried out at room temperature. Both uterine horns from an individual animal were processed together in the same vials to mitigate inter-batch variability.

#### Histology

After micrographs of CGRP-immunoreactivity were captured (see below), uterine preparations were unmounted from slides. Each uterine horn was segmented circumferentially into nine approximately equal tissue pieces, corresponding to the three image sections each taken from the cranial (ovarian), mid, and caudal (cervical) regions. Tissues were then placed in cassettes and submerged into 70% ethanol, then dehydrated in graded alcohols, cleared with xylene, and embedded with warmed liquid paraffin wax. Once solidified, circumferential sections of 5 μm were cut in triplicate using a rotary microtome and collected onto glass slides. Routine hematoxylin and eosin staining was then performed.

### *In vivo* retrograde tracing from uterus to dorsal root ganglia

#### Surgery

The procedure for injecting mouse uterus with retrograde neuronal tracer *in vivo* has also been described previously (Herweijer et al., 2014; Dodds et al., 2021). A separate cohort of mice (*N* = 4) underwent a 10 mm midline laparotomy under inhaled isoflurane anesthesia (induced at 4%; maintained at 1.5–2% in 1 L/min O_2_). Internal organs were reflected to expose the uterus, which was isolated from adjacent tissues by sterile saline-soaked gauze. Approximately 10 μL of the retrograde neuronal tracer, DiI (1,1′-didodecyl 3,3,3′,3′-tetramethylindocarbocyanine perchlorate; 2 mg/mL in dimethylformamide; Molecular Probes #D383; Eugene, OR, United States), was injected unilaterally into the caudal third of the uterine horn myometrium. DiI was delivered using a custom-made spritz system (Biomedical Engineering; Flinders University of South Australia; Bedford Park, SA, Australia) that applies pulses of nitrogen to a glass micropipette (inner diameter ∼5 μm; World Precision Instruments #TW150-4; Sarasota, FL, United States) for 1 s duration at 0.3 Hz, 10–15 psi, for periods of 5–10 min. Following injection, surrounding tissues were inspected for any dye leakage, rinsed with sterile saline, and the wound sutured closed. Animals received an identical peri- and post-operative drug and recovery schedule as described for the DRG removal procedure.

#### Tissue processing

After 7 days recovery, mice were euthanized by isoflurane overdose (5% in oxygen) and the heart removed. Thoracic (T10) to sacral (S2) DRG (inclusive) were dissected and fixed overnight in 4% PFA. DRG were then rinsed with PBS and mounted on glass slides with 100% carbonate-buffered glycerol.

### Image capture and analysis

For CGRP immunoreactivity, slides were viewed with an Olympus IX71 epifluorescence microscope (Shinjuku-ku, Tokyo, Japan) equipped with highly discriminating filters (Chroma Technology; Battledore, VT, United States). Micrographs were captured at 4x magnification (500 ms exposure; 1,392 × 1,040 pixels; field of view dimensions 2.249 × 1.680 mm^2^) using a CoolSNAP™ camera (Roper Scientific; Tucson, AZ, United States) and AnalySIS Image 5.0 software (Olympus-SIS; Münster, Germany), and saved as TIFF files. A total of nine images were systematically collected from the cranial- to caudal-end per uterine horn, with three files corresponding to each third of the uterine horn designated per region.

Background autofluorescence in each micrograph was normalized using the *Subtract Background* feature of Fiji Image J 1.52p software^1^ (RRID:SCR_002285) (Schindelin et al., 2012) with the rolling ball radius set to 50.0 pixels. To determine CGRP density (%) code written in PyCharm Community Edition 2019.3.4 software^2^ (RRID:SCR_018221) analyzed TIFF files in 8-bit grayscale and calculated the average overall pixel intensity of each micrograph, where total black pixels = 0% (immunoreactivity absent) and total white pixels = 100% (immunoreactivity present). All subsequent CGRP-expressing neuronal characteristics were calculated in Fiji Image J software. Fibers were quantified by counting axons that crossed an oblique transect line spanning the full length of each contrast-optimized micrograph (De Fontgalland et al., 2008). Fiber network lengths, junctions, and proportions of area containing CGRP immunoreactivity were then quantified in binarized micrographs after applying the Li-threshold. First, values for area of CGRP (%) were obtained by executing the *Area Fraction* measure. Skeletons were then extracted from micrographs using the *Skeletonize3D* plugin and analyzed with *AnalyzeSkeleton* (Arganda-Carreras et al., 2010). Nerve fiber network lengths (mm) were calculated as the sum of # end point voxels, # junction voxels, and # slab voxels, then converted into appropriate units. Total number of junctions were calculated as the sum of # junctions.

For histological sections, brightfield micrographs were captured at 40x magnification with an Olympus VS200 slide scanner (Shinjuku-ku, Tokyo, Japan). Myometrial thickness was assessed by manually placing the *Arbitrary Line* measurement tool over the appropriate tissue layers using VS200 Desktop 3.2.1 software (Olympus-SIS; Münster, Germany) (Supplementary Figure 1). Average thicknesses were calculated from three measurements of a given tissue segment. CGRP density was then corrected for myometrial thickness by dividing the density of immunolabeling by a “thickness ratio” (corresponding average regional thickness divided by average of all thickness values) in Microsoft^®^ Excel version 2204 (Microsoft Corporation; Redmond, WA, United States). From the three images taken per uterine horn region, a single mean CGRP density value (%) corrected for myometrial thickness was calculated.

Retrogradely-traced DiI-labeled cell somata in DRG were assessed using micrographs captured at 10x magnification (1,000 ms exposure) with an Olympus IX71 epifluorescence microscope, as described above. Multiple images were taken throughout the depth of each wholemount ganglion to ensure identification of all positively labeled cells. Background autofluorescence was determined per image by quantifying average fluorescence intensity in DRG over six random locations with a circular region of interest of 300 μm^2^. DiI-labeled cells were considered positive where punctate staining occurred with a fluorescence intensity > 2 SD above mean background autofluorescence.

### Statistics

Data are expressed as mean ± standard deviation (SD). “*N*” values refer to the number of animals, and “*n*” values the number of individual observations. All statistical analyses were performed in Prism 8 version 8.4.3 (GraphPad Software, Inc.; La Jolla, CA, United States). Statistical significance was accepted for *P* values < 0.05. CGRP density, nerve fiber counts, network lengths, junctions, and area of CGRP, were compared using a two-way, repeated measures ANOVA with Geisser-Greenhouse correction and Tukey’s multiple comparisons (fixed effect of region: cranial/mid/caudal uterine horn) or Šídák’s multiple comparisons (fixed effect of lesion side: ipsilateral/contralateral to DRG removal). Independent, non-surgical control data of CGRP density in the mouse uterine horn (*N* = 5) was originally published in Kyloh et al. (2022), in which the tissue had been processed identically to DRG-removed animals here. These control data were compared to values from DRG-removed animals using a mixed-effects model (REML) with Tukey’s multiple comparisons. Counts of thoracolumbar (T13-L2) versus lumbosacral (L5-S1) DRG cell somata retrogradely traced from the caudal uterine horn were compared using a paired, two-tailed *t* test.

## Results

**FIGURE 1:**
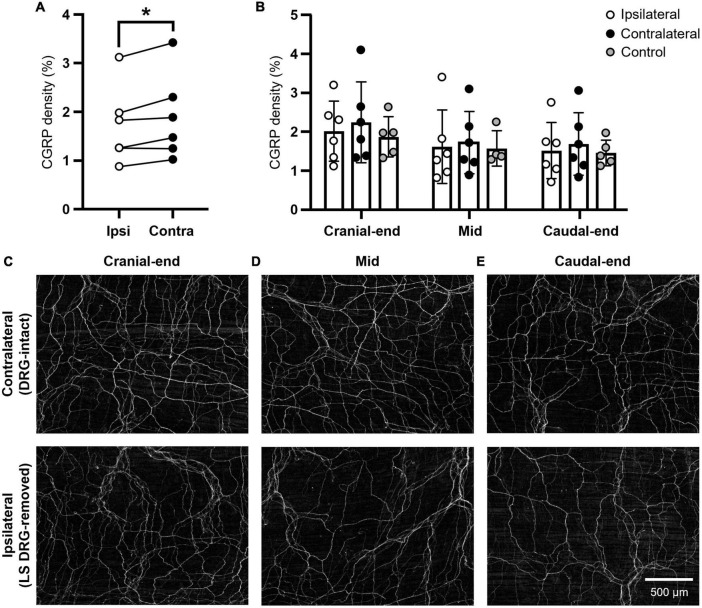
Density of CGRP immunoreactivity in the mouse uterine horn following unilateral lumbosacral L5-S1 DRG removal. **(A)** Summary data of paired, average CGRP density values across the whole uterine horn following unilateral lumbosacral L5-S1 removal. A significant reduction in CGRP immunoreactivity was observed in the ipsilateral (DRG-removed) horn compared to the contralateral (DRG-intact) side (*P* = 0.0265; two-way, repeated measures ANOVA with Šídák’s multiple comparisons; *N* = 6 per group). **(B)** Summary data of individual, average CGRP density values from each ipsilateral and contralateral uterine horn subregion (cranial-end, mid, caudal-end; *N* = 6 per group) and an independent, non-surgical control (*N* = 5). No regional differences in CGRP immunoreactivity were observed between the ipsilateral and contralateral uterine horns or compared with control values (mixed-effects model (REML) with Tukey’s multiple comparisons). **(C)** Representative images of CGRP immunoreactivity from the cranial uterine horn contralateral (upper) and ipsilateral (lower) to unilateral lumbosacral L5-S1 DRG removal in the same animal. **(D)** Representative images from the mid uterine horn. **(E)** Representative images from the caudal uterine horn. Scale bar in panel [**(E)**; lower] applies to all images. Independent control data in panel **(B)** was originally published in Kyloh et al. (2022). LS, lumbosacral; DRG, dorsal root ganglia; Ipsi, ipsilateral; Contra, contralateral; CGRP, calcitonin gene-related peptide.

CGRP density in the mouse uterine horn displayed intrinsic regional variation [*P* = 0.0002; F (1.8, 9.0) = 27.8; *N* = 6 per group]; most immunoreactivity concentrated in the cranial end, compared to the caudal region (ipsilateral *P* = 0.0043; contralateral *P* = 0.0082). Overall, density values were significantly reduced across the uterine horn ipsilateral to lumbosacral (L5-S1) DRG removal, compared to the contralateral side [*P* = 0.0265; F (1, 5) = 9.69; *N* = 6 per group] (Figure 1A). Interestingly, no statistical differences were observed in the cranial (ipsilateral: 2.0 ± 0.8%; contralateral 2.2 ± 1.0%; *P* = 0.4340) (Figures 1B,C), mid (ipsilateral: 1.6 ± 0.9%; contralateral: 1.7 ± 0.8%; *P* = 0.7312) (Figures 1B,D), or caudal (ipsilateral: 1.5 ± 0.7%; contralateral: 1.7 ± 0.8%; *P* = 0.2115) (Figures 1B,E) regions of the uterine horn. These values represent modest reductions in CGRP expression of 10, 6, and 10%, respectively. Inclusion of an independent, non-surgical control group (*N* = 5) also showed no significant differences in CGRP density to either the ipsilateral or contralateral uterine horns (*P* ≥ 0.7274 for all comparisons) (Figure 1B).

**FIGURE 2:**
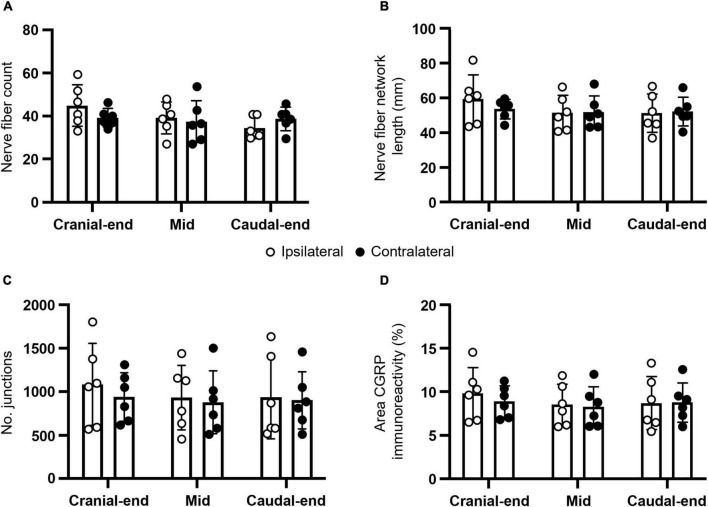
Anatomical characteristics of CGRP-expressing nerve fibers in the mouse uterine horn following unilateral lumbosacral L5-S1 DRG removal. Summary data of **(A)** nerve fiber counts; **(B)** nerve fiber network lengths; **(C)** number of junctions; and **(D)** proportion of area containing CGRP immunoreactivity. No statistically significant differences were observed between ipsilateral and contralateral uterine horns from DRG-removed animals for all measures (*N* = 6 per group). All panels were analyzed using two-way, repeated measures ANOVA with Šídák’s multiple comparisons. CGRP, calcitonin gene-related peptide; DRG, dorsal root ganglia.

In addition to total density, CGRP-expressing nerve fiber counts, network lengths, junctions, and proportions of area containing CGRP immunoreactivity were analyzed (Figure 2 and Table 1). No statistically significant main effects (*P* ≥ 0.2438) or regional *post hoc* differences (*P* ≥ 0.1527) were observed between ipsilateral and contralateral uterine horns from DRG-removed animals for all measures (*N* = 6 per group). However, normalized ipsilateral nerve fiber counts were 114 ± 22% of the contralateral uterine horn in the cranial region, 106 ± 15% in mid, and 90 ± 11% in the caudal region; consistent with the overall reduction in CGRP density of ∼10%, at least in the caudal uterine horn. Moreover, similar to CGRP density, nerve network lengths [*P* = 0.0157; F (1.8, 8.9) = 7.1] and area of CGRP immunoreactivity [*P* = 0.0291; F (2.0, 9.9) = 5.2] measures in the uterine horn were intrinsically greater in the cranial region.

**TABLE 1:** Measurements of CGRP-expressing uterine nerve fiber characteristics following lumbosacral DRG removal.

Uterine region	Nerve fiber count		Nerve fiber network		No. junctions		Area CGRP	
			length (mm)				immunoreactivity (%)	
				
	Ipsi	Contra	Ipsi	Contra	Ipsi	Contra	Ipsi	Contra
Cranial	44.8 ± 9.7	39.4 ± 4.2	59.8 ± 14.0	54.1 ± 5.6	1112.4 ± 487.5	964.8 ± 288.6	9.8 ± 3.0	8.9 ± 1.8
Mid	39.1 ± 7.5	37.4 ± 9.6	51.4 ± 10.0	51.7 ± 9.3	956.7 ± 382.0	899.5 ± 364.4	8.5 ± 2.4	8.3 ± 2.3
Caudal	34.4 ± 5.0	38.7 ± 5.4	51.1 ± 11.1	52.0 ± 8.3	958.5 ± 493.4	924.0 ± 341.3	8.7 ± 3.0	8.8 ± 2.3

Ipsi, ipsilateral; Contra, contralateral.

Studies in rat suggest the lower female reproductive tract (including the very caudal uterine horn, uterine cervix, and vagina) receives greater sensory innervation from lumbosacral inputs than the upper female reproductive tract (Berkley et al., 1988, 1990, 1993; Nance et al., 1988). Additionally, around half of CGRP-expressing nerve fibers persisted in the caudal uterine horn following thoracolumbar T13-L2 DRG removal (Kyloh et al., 2022). Hence, marked CGRP-expressing nerve depletion in the caudal uterine horn was expected following lumbosacral L5-S1 DRG removal, compared to the cranial and mid regions. Since no regional differences in CGRP density or other nerve characteristics were observed post-lumbosacral DRG removal, a separate series of retrograde neuronal tracing experiments were performed to determine the proportions of thoracolumbar and lumbosacral spinal afferents innervating the caudal region of the mouse uterine horn.

**FIGURE 3:**
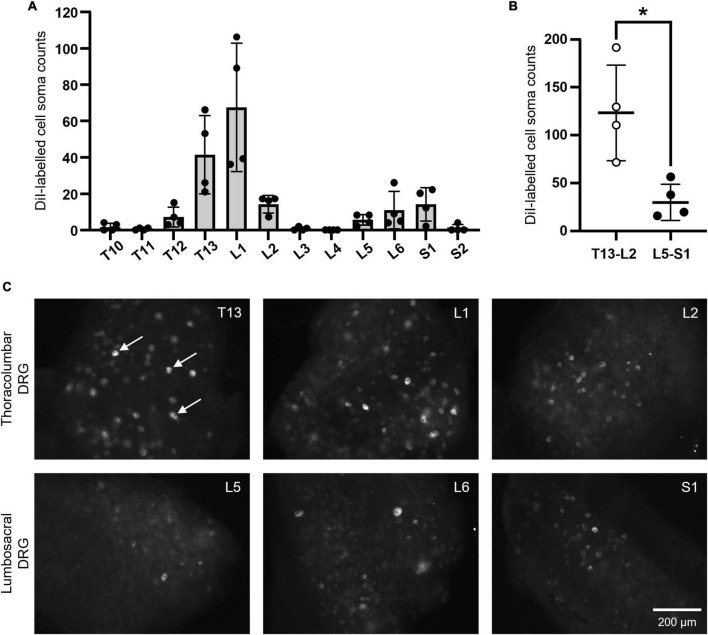
DiI-labeled somata in thoracolumbar and lumbosacral DRG following retrograde neuronal tracing from the caudal mouse uterine horn. **(A)** Summary data showing the distribution of *n* = 660 positively labeled cell soma counts across T10-S2 DRG (*N* = 4). **(B)** Combined values from thoracolumbar T13-L2 and lumbosacral L5-S1, demonstrating that thoracolumbar spinal afferents supply significantly greater innervation to the caudal uterine horn compared to lumbosacral spinal afferents (*P* = 0.0442; paired, two-tailed *t* test). **(C)** Representative images of somata that were retrogradely-labeled with DiI in thoracolumbar T13-L2 DRG (upper row) and lumbosacral L5-S1 DRG (lower row). Examples of somata considered to be positively labeled in the given focal plane are indicated by the arrows in panel T13 **(C)**. Scale bar in panel S1 **(C)** applies to all images. DRG, dorsal root ganglia.

A total of *n* = 660 retrogradely-labeled cell somata were identified across T10-S2 DRG ipsilateral to DiI injection of the caudal uterine horn (*N* = 4) (Figure 3A). Most labeling occurred within thoracolumbar T13-L2 DRG (*n* = 123.3 ± 50.0 somata) compared to lumbosacral L5-S1 DRG (*n* = 29.8 ± 18.8 somata) (*P* = 0.0442) (Figures 3B,C).

## Discussion

This study describes the extent of lumbosacral spinal afferent innervation to the mouse uterine horn, following unilateral L5-S1 DRG removal *in vivo*. The key advantage of using this technique is that we have been able to selectively deplete only spinal afferent neurons from lumbosacral DRG, as opposed to all autonomic sensory and motor pathways within the major nerve trunks (hypogastric and pelvic) that innervate the mouse uterus. In addition, unilateral DRG removal allows for the affected uterine horn to be compared against an internal control (the contralateral horn), given the lateralization of innervation supplied to this organ and the greater female reproductive tract (Klein and Burden, 1988; Nance et al., 1988). Here, we demonstrate that CGRP-expressing lumbosacral spinal afferent neurons supply relatively minor innervation across the mouse uterine horn. Subsequent retrograde neuronal tracing confirmed that few lumbosacral spinal afferent neurons innervate the caudal uterine horn, with a greater proportion arising from thoracolumbar DRG; consistent with tracing from the mid and whole uterus (Herweijer et al., 2014; Dodds et al., 2021). Collectively, these findings suggest that sensory information between the mouse uterine horn and central nervous system is primarily relayed *via* thoracolumbar spinal afferent neurons.

Previous anatomical and functional studies on the rat female reproductive tract suggested that its sensory innervation follows a distinct topographical (though overlapping) arrangement, with the upper portion predominantly supplied by thoracolumbar DRG *via* the hypogastric nerves and the lower portion by lumbosacral DRG *via* the pelvic nerves (Berkley et al., 1988, 1990, 1993; Nance et al., 1988). The latter included the caudal uterine horn, below the internal bifurcation. Hence, we expected to observe a more pronounced depletion of CGRP-expressing spinal afferent fibers in this region of the mouse uterine horn following lumbosacral L5-S1 DRG removal, compared to the cranial and mid portions. While there did appear to be a subtle loss of nerve fiber numbers in the caudal region, our CGRP density data indicate a relatively uniform distribution of lumbosacral spinal afferent innervation across the mouse uterine horn; albeit only modest compared with that supplied by thoracolumbar T13-L2 DRG.

This finding indicates a possible species difference in the distribution of lumbosacral spinal afferent innervation to the uterine horns between female rodents. For future studies on uterine sensory innervation, this distinction may be important; given the growing utility of genetically modified mouse models in understanding biological systems. In any case, the present study replicated a separate finding from the rat, in that CGRP-expressing nerve fibers were intrinsically more concentrated within the cranial region of the uterine horn, compared to the middle and caudal portions (Zoubina et al., 1998). Use of CGRP as a sensory marker is in line with our recent study that identified spinal afferent nerve endings in the mouse uterus for the first time (Dodds et al., 2021). Other neurochemical markers that label sensory neurons (such as substance P) may reveal a different pattern innervation to the mouse uterine horn following DRG removal.

Nonetheless, when considered together with our initial study describing the unilateral DRG removal technique (Kyloh et al., 2022), thoracolumbar and lumbosacral spinal afferents account for ∼70% of all CGRP immunoreactivity in the cranial uterine horn, and ∼55% in the caudal uterine horn. Although these values represent the majority of this neurochemical class, the origin and identity of the residual CGRP-expressing neurons requires deeper investigation. Estrous cycle variability in the expression of CGRP is unlikely, as this factor was controlled for *via* myometrial thickness correction and has been reported as negligible in other rodent uteri (Zoubina et al., 1998). Rather, these CGRP-expressing fibers probably arise from spinal afferent somata in DRG adjacent to thoracolumbar T13-L2 and lumbosacral L5-S1, and vagal afferents. Some contribution from contralateral DRG may be possible; however, unilateral anterograde tracing from thoracolumbar DRG only resulted in labeled fibers in the ipsilateral uterine horn of rats (Nance et al., 1988) and mice (Dodds et al., 2021). Thus, reinforcing the concept of a lateralized neuronal input into each half of the female reproductive tract. Our studies using DRG removal may also underestimate the true extent of thoracolumbar- and lumbosacral-associated CGRP depletion in the uterine horn, where there is potential for postsurgical compensatory changes in CGRP-immunoreactivity induced by remaining neuronal populations (Kyloh et al., 2022), as has been observed for cutaneous afferents in models of spared nerve injury (Duraku et al., 2012; Nascimento et al., 2015; Yeh et al., 2021). Although, we recently reported that 6 months after lumbosacral DRG removal, electrical stimulation of the rectum or bladder was unable to elicit any visceromotor response (VMR) (Kyloh et al., 2022).

The physiological purpose for having dual thoracolumbar and lumbosacral spinal afferent innervation to the uterus, and indeed other pelvic viscera, is not fully understood. One hypothesis is that each division detects and conveys modality-specific sensory information. In line with data on the colorectum (Wang et al., 2005; Christianson et al., 2006; Harrington et al., 2019), earlier functional studies on the rat female reproductive tract suggested that hypogastric and pelvic nerve fibers have different sensitivities to mechanical and chemical stimuli (Berkley et al., 1988, 1990, 1993), which may alter across the reproductive cycle and under pathophysiological conditions (Robbins et al., 1990, 1992; Temple et al., 1999). Collectively, these response properties indicate that pelvic nerve activity aligns with behavioral processes associated with mating and conception; the hypogastric nerves contribute to pregnancy and nociception; with both nerve pathways involved in parturition (Peters et al., 1987; Berkley et al., 1993). Our DRG removal technique thus offers a new approach to further discriminate how these sensory nerves communicate between the uterus and central nervous system, by allowing direct interrogation of specific spinal afferent pathways, or vagal afferent pathways devoid of spinal afferents.

## Concluding remarks

The present study has demonstrated that CGRP-expressing lumbosacral spinal afferent neurons supply relatively modest sensory innervation across the mouse uterine horn. This was achieved using a surgical technique in mice recently developed by our laboratory, where select DRG of interest (in this case, lumbosacral L5-S1) are unilaterally removed *in vivo*. Retrograde neuronal tracing from the caudal uterine horn confirmed that few lumbosacral spinal afferents innervate this region, with most uterine nerve fibers instead supplied by thoracolumbar T13-L2 DRG. This quantitative data provides foundational knowledge for understanding the anatomy of sensory pathways in the mouse uterus, which is paramount before we can fully interpret studies of uterine biology using genetically modified mouse models. Our DRG removal technique may continue to be used in future works to decipher the specific spinal pathways by which sensory information, including pain-associated stimuli, is relayed between the uterus (and other visceral organs) and the central nervous system.

## Data availability statement

The raw data supporting the conclusions of this article will be made available by the authors, without undue reservation.

## Ethics statement

This animal study was reviewed and approved by Animal Welfare Committee of Flinders University of South Australia (approval #966/19).

## Author contributions

KD and NS conceived and designed research. MK developed and employed the technique to surgically remove DRG from live mice. KD, MK, and LT performed experiments. KD, MC, and TH analyzed data. KD, TH, and NS interpreted results of experiments. KD prepared figures and drafted manuscript. All authors edited and revised manuscript and approved final version of manuscript.

## Footnotes

1
www.fiji.sc

2
www.jetbrains.com/pycharm

## Supplementary material

Supplementary Figure 1 Histology of the uterine myometrium. **(A)** Example low-power view of a cross section through the mid uterine myometrium. **(B)** Expanded image of box in panel **(A)**. The Arbitrary Line tool in VS200 Desktop 3.2.1 software was used to measure total myometrial thickness. Average thicknesses calculated over three sections were then used to generate a “thickness ratio” (average regional thickness divided by average of all thickness values) to correct corresponding CGRP density values.
