# Supplementary figures and images for: Anatomical distribution of CGRP-containing lumbosacral spinal afferent neurons in the mouse uterine horn
Source: Front Neurosci. 2022 Sep 28;16:1012329. doi: 10.3389/fnins.2022.1012329 (PMC9554138; doi:10.3389/fnins.2022.1012329)

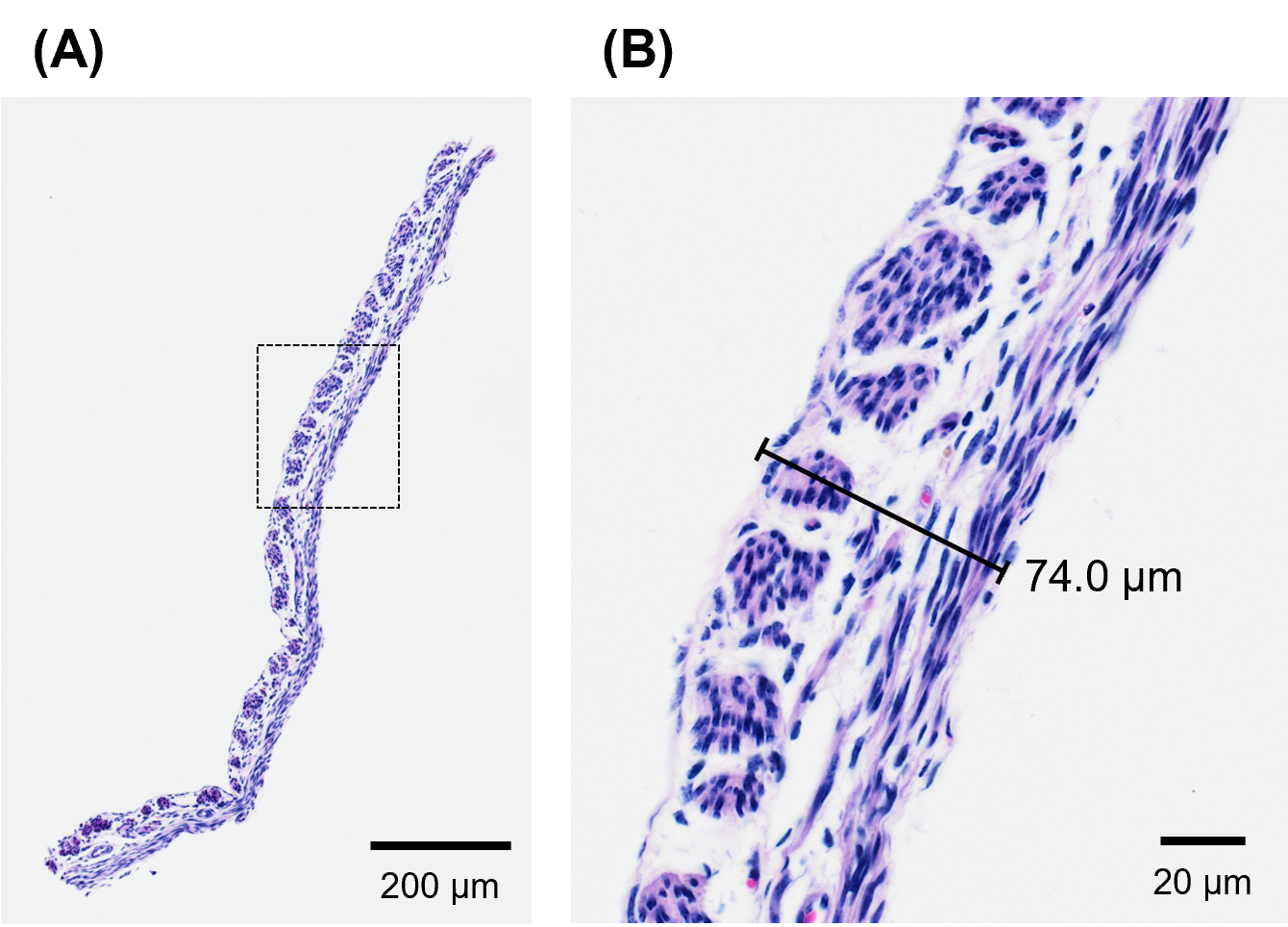

Supplement: Supplementary Figure 1 — Histology of the uterine myometrium. (A) Example low-power view of a cross section through the mid uterine myometrium. (B) Expanded image of box in panel (A). The Arbitrary Line tool in VS200 Desktop 3.2.1 software was used to measure total myometrial thickness. Average thicknesses calculated over three sections were then used to generate a “thickness ratio” (average regional thickness divided by average of all thickness values) to correct corresponding CGRP density values. [file Image_1.TIF]
